# Cerebellar Structural Abnormalities Associated With Cognitive Function in Patients With First-Episode Psychosis

**DOI:** 10.3389/fpsyt.2018.00286

**Published:** 2018-07-03

**Authors:** Taekwan Kim, Kwang-Hyuk Lee, Hyerim Oh, Tae Young Lee, Kang Ik K. Cho, Junhee Lee, Jun Soo Kwon

**Affiliations:** ^1^Department of Brain and Cognitive Sciences, College of Natural Sciences, Seoul National University, Seoul, South Korea; ^2^Department of Neuropsychiatry, Seoul National University Hospital, Seoul, South Korea; ^3^Institute of Human Behavioral Medicine, Medical Research Center, Seoul National University, Seoul, South Korea; ^4^Department of Psychiatry, College of Medicine, Seoul National University, Seoul, South Korea

**Keywords:** schizophrenia, FEP, cerebellum, neurocognition, executive function, neuroimaging, SUIT

## Abstract

**Introduction:** The fundamental role of the cerebellum in higher cognitive processing has recently been highlighted. However, inconsistent findings exist in schizophrenia with respect to the exact nature of cerebellar structural abnormalities and their associations with cognitive and clinical features.

**Materials and Methods:** We undertook a detailed investigation of cerebellar lobular volumes in 40 patients with first-episode psychosis (FEP) and 40 healthy controls (HCs) using the spatially unbiased atlas template of the cerebellum (SUIT). We examined the functional significance of cerebellar structural abnormalities in relation to cognitive and clinical outcomes in patients.

**Results:** We found that left cerebellar lobules VI and X volumes were lower in FEP patients, compared to HCs. Smaller left lobules VI and X volumes were associated with fewer number of categories completed on the Wisconsin Card Sorting Test (WCST) in patients. In addition, smaller left lobule X volume was related to performance delay on the Trail Making Test (TMT) Part B in patients.

**Conclusion:** Our results demonstrate that cerebellar structural abnormalities are present at the early stage of schizophrenia. We suggest functional associations of cerebellar structural changes with non-verbal executive dysfunctions in FEP.

## Introduction

Increasing evidence suggests that the cerebellum is associated with cerebral networks involved in cognitive and emotional processes, as well as motor control (1–3). Neuropsychological studies have shown that the cerebellum is associated with major neurocognitive domains such as processing speed, working memory, verbal fluency, and executive function (4–7). Neuroimaging studies have further shown that the lateral hemispheres of the posterior cerebellum (lobules VI–IX) and flocculonodular lobe (lobule X) were associated with cognitive functions, while the anterior lobe (lobules I-V) was primarily responsible for motor functions (2, 8–10). Although the aforementioned neurocognitive domains are known to be impaired in schizophrenia (11), it is still not fully understood how these neurocognitive domains are associated with specific cerebellar volumetric abnormalities in schizophrenia.

To date, the majority of neuroimaging studies in schizophrenia have reported some aspects of cerebellar abnormalities, although they are mostly inconsistent. A smaller whole cerebellar volume has been reported (12–14), but not replicated (15–17). In fact, Wilke and colleagues have even reported larger cerebellar volume in patients (18). Of studies seeking specific cerebellar regional volume deficits in schizophrenia, smaller volumes in the posterior superior lobe, such as lobule VI and Crus I/II, have been reported (19–22). Other studies have also reported smaller volume in the anterior or posterior inferior lobe, including lobule X (20, 23–25). Yet again, some studies found larger or no different volume in such regions (26–29). Relatively few studies have investigated relationships between cerebellar volumetric differences and neurocognitive deficits in schizophrenia with varying cerebellar foci (21, 30–32). Hence, it is yet to be determined whether cerebellar structure-function relationships are present in schizophrenia.

Potential reasons for this inconsistency in cerebellar structural findings in schizophrenia include the use of different methodological procedures to analyze cerebellar volumes. One of three methodologies has been used to extract cerebellar volumes: manual delineation, automated segmentation based on image normalization to whole-brain template, or automated segmentation using an isolated cerebellar image. In the absence of automated segmentation methods, the manual delineation method was used to measure cerebellar volumes (23, 25, 33). Later, researchers segmented cerebellar tissues after registering brain images into a whole-brain standard reference such as the Montreal Neurological Institute (MNI) template (34). This was widely adopted into the whole-brain voxel-based morphometry (VBM) method (35) and FreeSurfer pipeline (36). However, it had an innate issue of poor alignment of cerebellar structures across individuals, which limited anatomical details of cerebellar lobules (37). Thus, it was suggested to use isolated cerebellar images and a cerebellum-specific template such as the spatially unbiased atlas template of the cerebellum (SUIT) to improve the overlap of cerebellar structures across participants (37–39). Hence, the cerebellum-specific methods are expected to produce more consistent findings for cerebellar structural abnormalities in schizophrenia.

One of the other reasons for these inconsistent findings includes the illness chronicity of patients (40–42). It is critical to understand whether cerebellar abnormalities are present at the early phase of this illness, or whether they are associated with illness progression. However, most previous studies used a chronic sample of schizophrenia patients, producing confounding effects due to relatively varying degrees of illness duration, age, and medication. Like studies of chronic patients, studies of first-episode psychosis (FEP) patients have reported that the overall cerebellar gray matter (GM) volume was smaller compared to healthy controls (HCs) (14, 22). Furthermore, longitudinal studies have shown a reduced GM volume in the right cerebellum in high-risk individuals who subsequently developed schizophrenia (43, 44). Although these studies support the hypothesis that cerebellar abnormalities are not a result of the adverse effects from chronic deteriorating course of the illness or medications, relationships between neurocognitive functions and cerebellar lobular abnormalities have not been investigated in FEP.

In the present study, we conducted a detailed investigation of cerebellar lobules in 40 FEP patients and 40 HCs using SUIT that retained anatomical details within infra-tentorial structures. We also wished to examine the functional significance of cerebellar structural abnormalities in relation to cognitive and clinical outcomes in patients compared to HCs. Using FEP subjects, instead of chronic patients, minimizes the confounding effects resulting from both illness chronicity and long-term antipsychotic medications on cerebellar structural abnormalities. We hypothesized that FEP patients would exhibit smaller volumes in cerebellar lobules, especially in the posterior lobe. In addition, these structural abnormalities would have correlations with clinical and neurocognitive function measures in patients.

## Materials and methods

### Participants

Forty patients with FEP were recruited between April 2010 and June 2016 from a prospective cohort study in the Seoul Youth Clinic (www.youthclinic.org), a center for early detection and intervention of individuals at high-risk for psychosis (45). The diagnosis of FEP was confirmed using the Structured Clinical Interview for DSM-IV Axis I Disorders, Patient Edition [SCID-I/P; (46)] by experienced psychiatrists. All patients had a history of less than 1 year since their first psychotic episode, which was defined as suffering from a brief psychotic disorder (*N* = 1), schizophreniform disorder (*N* = 11), schizoaffective disorder (*N* = 3), schizophrenia (*N* = 24), or psychotic disorder, not otherwise specified (*N* = 1), according to the DSM-IV-TR criteria. Twenty-nine patients were treated with antipsychotic medications, while others were not being medicated. We administered the Positive and Negative Syndrome Scale (PANSS) (47) and the Global Assessment of Functioning (GAF) (48) to assess symptom severity and overall functioning of patients. Forty HCs were recruited through internet advertisements (Table 1) and screened with SCID-I, Non-Patient Version [SCID-I/NP; (49)]. Exclusion criteria for all participants were brain injury or neurological disorders, major psychiatric disorders other than psychotic disorders, substance abuse, or Intelligence Quotient (IQ) below 70. After a complete description of the study was provided to participants, written informed consent was obtained. The Institutional Review Board of Seoul National University Hospital approved this study.

**Table 1:** Demographic and clinical results.

**Variables**	**FEP (*N* = 40)**	**HCs (*N* = 40)**	**Statistics**	
	***N***		***X*^2^ (*df*)**	***p***
Sex (male/female)	18/22	19/21	0.05 (1)	0.823
Handedness (right/left)	34/5	39/1	3.00 (1)	0.083
	**Mean (*****SD*****)**		***T*** **(*****df*****)**	***p***
Age (years)	22.9 (5.64)	23.13 (5.03)	0.19 (78)	0.851
IQ	98.33 (13.13)	115.68 (10.91)	6.39 (77)	< 0.001
TIV (L)	1.57 (0.18)	1.57 (0.16)	−0.05 (78)	0.958
CPZ equivalent dose (mg/day)	282.89 (272.49)			
Duration of illness (years)	0.53 (0.39)			
GAF	45.95 (9.66)			
GAF past year	70.13 (11.86)			
**PANSS**				
Positive	16.55 (4.86)			
Negative	17.6 (5.47)			
General	35.43 (7.23)			
Total	69.58 (13.64)			

^*^FEP, first-episode psychosis; HCs, healthy controls; TIV, total intracranial volume; CPZ, chlorpromazine; GAF, global assessment of functioning; PANSS, positive and negative syndrome scale.

### Neurocognitive function tests

Estimated IQ was obtained using the short form of the Korean version of the Wechsler Adult Intelligence Scale (WAIS) (50). A battery of neurocognitive function tests was used to assess the following neurocognitive domains in all participants: for cognitive processing speed, the Trail Making Test (TMT) Part A was used (51); visuospatial memory was assessed using the Rey-Osterrieth Complex Figure Test (RCFT), consisting of immediate and delayed recall stages (52); verbal executive function was assessed in letter and category fluency tasks using the Korean version of the Controlled Oral Word Association (COWA) test (53, 54); and the TMT Part B and the Wisconsin Card Sorting Test (WCST) were administered to assess non-verbal executive function (51, 55).

### Image acquisition and processing

All participants were scanned with a 3T Trio magnetic resonance imaging (MRI) scanner (Siemens Magnetom Trio, Erlangen, Germany) using a 12-channel head coil at Seoul National University Hospital. The T1-weighted anatomical image was acquired using magnetization-prepared rapid gradient echo (MPRAGE) imaging (echo time [TE]/repetition time [TR] = 1.89/1670 ms, field of view [FOV] = 250 mm, flip angle = 9°, matrix = 256 × 256, voxel size = 1.0 × 1.0 × 1.0 mm^3^, 208 slices). The time required to acquire the T1 image was 234 s.

Structural T1 images were preprocessed using the SUIT toolbox (37) implemented in the Statistical Parametric Mapping toolbox version 12 (SPM12; http://www.fil.ion.ucl.ac.uk/spm/). The images were segmented into GM, white matter (WM), and cerebrospinal fluid (CSF) using the unified segmentation algorithm (56). The cerebellum and brainstem were then isolated and normalized into a study-specific template using the Dartel algorithm that applied tissue segmentation maps (57, 58). Lastly, the images were resliced into the SUIT space using flow field and affine transformation matrix.

Based on the structural images registered into the SUIT space, we calculated volumes of all 28 cerebellar lobules. In addition to the SUIT processing, T1-weighted images were processed using FreeSurfer v.5.3.0 to obtain cerebellar hemispheric GM/WM volumes and total intracranial volume (TIV) (59).

### Statistical analyses

All statistical analyses were performed using SPSS, version 23 (IBM, Armonk, N.Y.). Demographics and neurocognitive function scores were compared between FEP and HCs using two-tailed independent samples *t-*test. To assess influence of IQ on neurocognitive function, we additionally conducted ANCOVA with IQ as a covariate for tests of neurocognitive differences.

One-way analysis of covariance (ANCOVA) with TIV as a covariate was conducted between FEP and HCs on cerebellar hemispheric and each of the 28 cerebellar lobular volumes. As previous studies suggested a possibility of sex differences in cerebellum abnormalities in schizophrenia (26, 60), we also conducted subgroup analyses of cerebellar lobular volume differences between FEP and HCs by sex. We addressed the problem of false positives during multiple comparisons based on the weighted multiple testing correction, which approximates multivariate normal distribution using correlation matrix (61, 62). The method of multiple comparisons correction assumes (1) that test statistics are asymptotically distributed as multivariate normal with known correlation matrix and (2) that the statistical power depends on the assigned weights and the extent of correlation among endpoints (62).

In each of the groups, we conducted partial correlation analyses between cerebellar volumes and neurocognitive functions, using TIV as a covariate. In the patient group, the partial correlations between cerebellar volumes and clinical outcomes were also analyzed with TIV as a covariate. We further conducted linear regression analyses with each neurocognitive function as a dependent variable, with group, cerebellar volume, TIV, and group-by-volume interaction term as predictors. The results of the analyses were not adjusted for multiple comparisons.

## Results

### Demographic and clinical characteristics

Demographic and clinical variables are shown in Table 1. There were no significant differences between patients and HCs for sex, age, or handedness. However, FEP patients had lower estimated IQ than HCs [*t*_(77)_ = 6.39, *p* < 0.001]. The two groups did not differ significantly in TIV.

### Neurocognitive functions

As shown in Table 2, FEP patients showed delayed reaction time (RT) in the TMT Part A [*t*_(64)_ = −4.54, *p* < 0.001] and Part B [*t*_(47)_ = −4.10, *p* < 0.001]. The accuracy of both immediate [*t*_(72)_ = 2.69, *p* < 0.01] and delayed recall [*t*_(69)_ = 3.44, *p* < 0.01] of the RCFT were lower in FEP patients. Both letter [*t*_(72)_ = 4.81, *p* < 0.001] and category [*t*_(72)_ = 6.02, *p* < 0.001] verbal fluency scores of the COWA were significantly lower in FEP patients. In addition, patients also had more perseverative errors [*t*_(49)_ = −2.49, *p* < 0.05] and less categories completed [*t*_(57)_ = 2.13, *p* < 0.05] on the WCST compared to HCs. All the neurocognitive functions, other than category fluency of the COWA [*F*_(1, 71)_ = 10.49, *p* = .002], were no longer significantly different when we adjusted IQ as a covariate.

**Table 2:** Comparisons of neurocognitive functions between FEP patients and HCs.

**Variables**	**FEP**	**HCs**	**Statistics**		
	**Mean (*****SD*****)**		***T* (df)**	***p***	**Cohen's *d***
**TMT**					
Part A RT	29.62 (9.94)	21.05 (6.16)	−4.54 (64)	<0.001	1.04
Part B RT	84.23 (45.25)	52.73 (15.64)	−4.10 (47)	<0.001	0.93
**RCFT**					
Immediate recall	11.49 (3.80)	13.63 (2.93)	2.69 (72)	<0.01	0.63
Delayed recall	11.26 (3.73)	13.86 (2.75)	3.44 (69)	<0.01	0.79
**COWA**					
Letter	32.72 (11.10)	44.6 (10.04)	4.81 (72)	<0.001	1.12
Category	31.46 (7.75)	42.91 (8.63)	6.02 (72)	<0.001	1.40
**WCST**					
Perseverative errors	13.15 (10.12)	8.83 (3.99)	−2.49 (49)	<0.05	0.56
Categories completed	5.44 (1.21)	5.9 (0.63)	2.13 (57)	<0.05	0.48

^*^FEP, first-episode psychosis; HCs, healthy controls; TMT, Trail Making Test; RCFT, Rey-Osterrieth Complex Figure Test; COWA, Controlled Oral Word Association Test; WCST, Wisconsin Card Sorting Test.

### Cerebellar hemispheric and lobular volumetric comparisons

**Figure 1:**
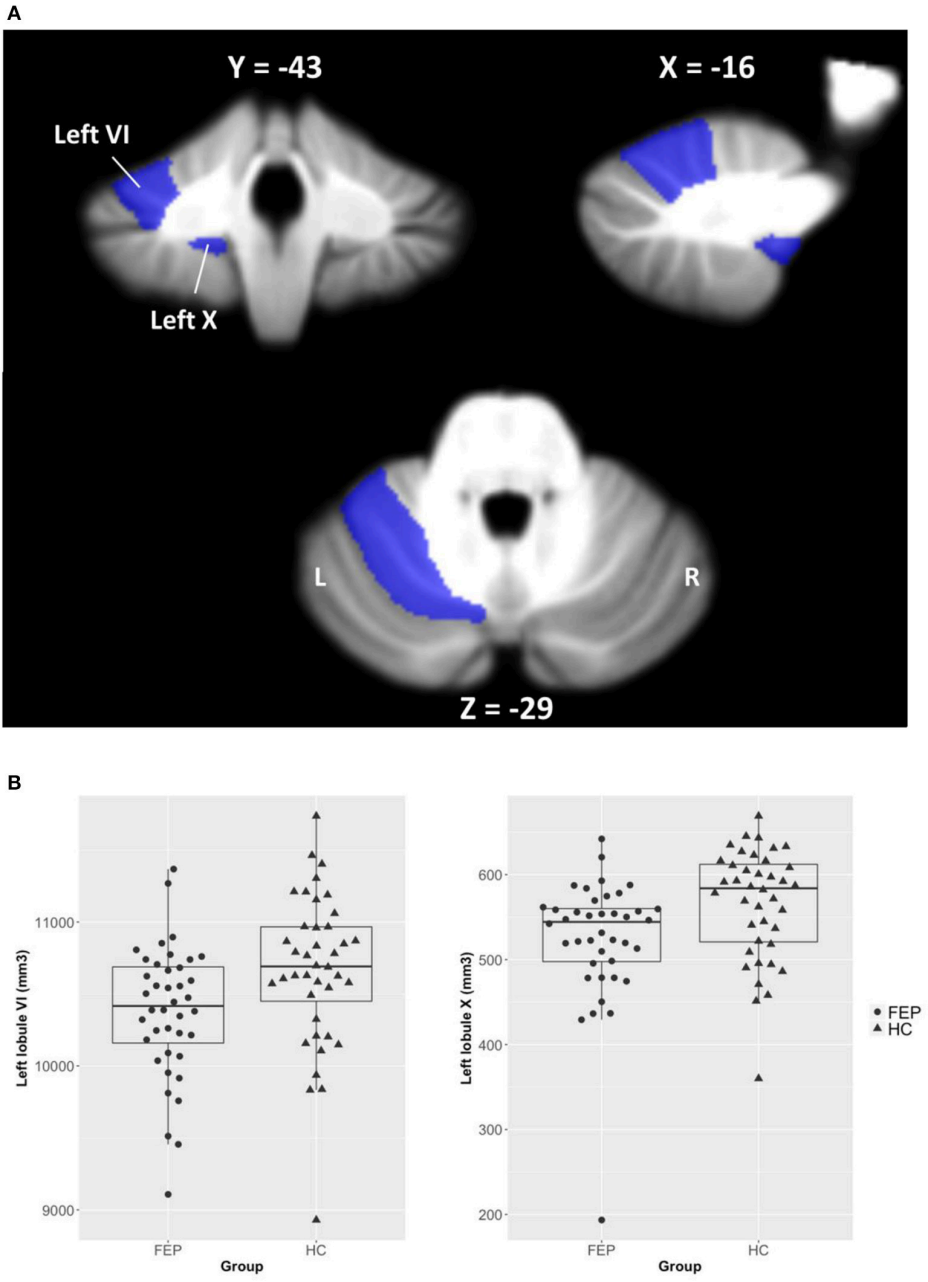
Volumetric differences of cerebellar lobules between FEP patients and HCs. **(A)** Regions of interest (ROIs) were selected from 28 cerebellar lobules using the SUIT cerebellum atlas, and ROIs of left lobules VI and X that significantly differed between groups are shown in the figure. **(B)** Cerebellar volumes of left lobules VI and X are smaller in FEP patients compared to HCs.

We found no group differences in left and right cerebellar hemispheric GM/WM volumes. As can be seen in Figure 1, we observed significantly smaller GM volumes in FEP patients in left lobule VI [*F*_(1, 77)_ = 6.66, corrected *p* < 0.05] and left lobule X [*F*_(1, 77)_ = 6.77, corrected *p* < 0.05], when controlling for TIV. Full details on between-group differences including trend level findings are presented in Table 3. There was a significantly smaller volume in vermis lobule VI [*F*_(1, 34)_ = 6.90, corrected *p* < 0.05] when comparing male subjects between patients and HCs, while none of the cerebellar lobules were different in female subjects.

**Table 3:** Differences in cerebellar lobular volumes between FEP and HCs.

**Lobules**	**FEP**	**HCs**	**Statistics**			
	**Mean (*****SD*****)**		***F***	***p***	**Corrected *p***	**η^2^**
**LEFT HEMISPHERE (cm**^3^**)**						
I-IV	3.04 (0.22)	3.10 (0.20)	2.02	0.159	1.000	0.03
V	4.61 (0.27)	4.70 (0.24)	2.47	0.120	0.993	0.03
VI	10.38 (0.46)	10.67 (0.53)	6.66	0.012	0.049	0.08
Crus-I	13.34 (0.72)	13.52 (0.65)	1.45	0.232	1.000	0.02
Crus-II	9.60 (0.57)	9.63 (0.34)	0.09	0.772	1.000	0.00
Vllb	5.07 (0.30)	5.17 (0.18)	3.25	0.075	0.895	0.04
Vllla	5.26 (0.29)	5.37 (0.21)	3.6	0.062	0.829	0.05
Vlllb	3.96 (0.36)	3.98 (0.22)	0.18	0.675	1.000	0.00
IX	2.86 (0.49)	3.02 (0.34)	2.77	0.100	0.969	0.04
X	0.52 (0.07)	0.57 (0.07)	6.77	0.011	0.046	0.07
**RIGHT HEMISPHERE (cm**^3^**)**						
I-IV	3.45 (0.19)	3.51 (0.21)	1.54	0.218	1.000	0.02
V	4.50 (0.21)	4.52 (0.23)	0.05	0.825	1.000	0.00
VI	9.18 (0.38)	9.34 (0.55)	2.4	0.125	0.996	0.03
Crus-I	13.56 (0.79)	13.94 (0.74)	4.93	0.029	0.535	0.06
Crus-II	9.35 (0.61)	9.53 (0.39)	2.37	0.128	0.998	0.03
Vllb	5.36 (0.34)	5.49 (0.19)	4.16	0.045	0.705	0.05
Vllla	4.96 (0.33)	4.99 (0.21)	0.26	0.613	1.000	0.00
Vlllb	3.92 (0.33)	3.91 (0.21)	0.06	0.813	1.000	0.00
IX	3.30 (0.42)	3.42 (0.37)	1.79	0.184	1.000	0.02
X	0.42 (0.08)	0.46 (0.06)	5.49	0.022	0.440	0.07
**VERMIS (cm**^3^**)**						
VI	1.78 (0.12)	1.84 (0.12)	5.05	0.028	0.522	0.06
Crus-I	0.01 (0.00)	0.01 (0.00)	0.27	0.608	1.000	0.00
Crus-II	0.42 (0.04)	0.42 (0.04)	0.03	0.872	1.000	0.00
Vllb	0.22 (0.02)	0.22 (0.02)	0.19	0.665	1.000	0.00
Vllla	1.25 (0.11)	1.26 (0.09)	0.03	0.856	1.000	0.00
Vlllb	0.60 (0.05)	0.61 (0.06)	0.56	0.457	1.000	0.00
IX	0.79 (0.07)	0.82 (0.07)	4.94	0.029	0.535	0.06
X	0.25 (0.02)	0.26 (0.02)	0.33	0.567	1.000	0.00

^*^FEP, first-episode psychosis; HCs, healthy controls.

### Correlation and regression analyses

Symptom severity was not associated with left lobe VI or X volume in patients. Smaller left lobule VI volume was associated with lower categories completed scores on the WCST in patients (*r* = 0.38, *p* < 0.05). Smaller left lobule X volume showed associations with delayed RT on the TMT Part B (*r* = −0.51, *p* < 0.01) and less categories completed on the WCST (*r* = 0.39, *p* < 0.05) within FEP patients. All the correlations observed in FEP patients were not found in HCs. Nonetheless, smaller left lobule VI volume was associated with delayed RT on the TMT Part A in HCs (*r* = −0.40, *p* < 0.05). Additional results of correlations between volumes of cerebellar regions (that produced non-significant between-group differences) and neurocognitive functions are presented in Tables S1, S2.

The results of regression analyses with HCs serving as a reference group are as follow. We found effects of group (*Beta* = −6.26, *p* < 0.01) and group-by-volume interaction of left lobule VI (*Beta* = 6.02, *p* < 0.05) on categories completed scores on the WCST, in which the interaction result indicated that FEP patients with larger left lobule VI volume had less impaired function in the WCST categories completed. The categories completed scores were also significantly predicted by group (*Beta* = −2.05, *p* < 0.05) and group-by-volume interaction of left lobule X (*Beta* = 1.84, *p* < 0.05) effects. In addition, group (*Beta* = 3.01, *p* < 0.001) and group-by-volume interaction of left lobule X (*Beta* = −2.61, *p* < 0.001) effects were variables that significantly predicted TMT Part B RT. The results of interaction terms of left lobule X indicated that FEP patients with larger volume of the region had less impaired functions in the WCST categories completed and TMT Part B. Full results of group-by-volume interaction effects of cerebellar regions (that produced non-significant between-group differences) on neurocognitive functions are presented in Table S3.

Within the patient group, volumes of left lobule VI and X were not significantly correlated with medication (chlorpromazine equivalent dose) (ρ = −0.28, *p* = 0.086; ρ = 0.03, *p* = 0.852), duration of illness (*r* = 0.04, *p* = 0.835; *r* = −0.22, *p* = 0.19), or IQ (*r* = −0.21, *p* = 0.214; *r* = 0.26, *p* = 0.115). However, age and duration of antipsychotic treatment had a significant negative correlation with left lobule VI (*r* = −0.36, *p* < 0.05; *r* = −0.34, *p* < 0.05), but not with left lobule X (*r* = −0.03, *p* = 0.876; *r* = −0.27, *p* = 0.103) volume.

## Discussion

The present study examined the volume of each cerebellar lobule using SUIT in FEP patients and HCs. Our findings confirmed cerebellar volumetric abnormalities in FEP patients. FEP patients had specific volume deficits in the left lobule VI of the posterior superior lobe and left lobule X of the flocculonodular lobe. Furthermore, we observed that smaller volumes in these cerebellar regions were significantly associated with non-verbal executive functions, measured by both the TMT and WCST. Thus, this study provides evidence for altered cerebellar morphology and its associations with neurocognitive deficits in the early phase of schizophrenia.

We found smaller cerebellar GM volume in left lobule VI of patients, which was consistent with previous findings in both FEP patients (20) and chronic schizophrenia (3). Our finding of smaller left lobule VI volume together with executive dysfunction in FEP is important because it is line with previous functional neuroimaging studies showing an association between left lobule VI and executive function (63). Furthermore, recent functional connectivity studies have indicated that lobule VI is part of the ventral attention network (VAN) implicated in stimulus-driven attentional control (1, 64, 65). The association between smaller left lobule VI volume and neurological soft signs (NSS), which are subtle neurological abnormalities associated with cognitive impairments (66), in schizophrenia also supports the role of left lobule VI in neurocognitive deficits in the patients (22, 67, 68). We suggest that smaller left lobule VI volume could lead to executive dysfunction due to a disruption of VAN and contribute to the generation of NSS in schizophrenia.

We observed smaller left lobule X volume, which was consistent with previous cerebellar structural studies with FEP (20) as well as with chronic schizophrenia patients (3). Lobule X (i.e., flocculonodular lobe) is phylogenetically the oldest portion of the cerebellum and coordinates balance and movement via the vestibular system (69–71). Contrary to the traditional concept of the vestibular system, recent studies have shown evidence that vestibular dysfunction is related to impairments in executive functions, such as attentional set-shifting and cognitive inhibition (72–74). For example, Bigelow et al. showed an association between vestibular function and performance on TMT Part B, which assesses attentional set-shifting (73). Performance on the Stroop Task, measuring cognitive flexibility and cognitive inhibition (75), was also associated with vestibular dysfunction (74). Furthermore, at the whole-brain level, lobule X is part of the default mode network (DMN) involved in attention and cognitive shifting (1, 3, 64, 76, 77). Consistent with this, Kansal and colleagues reported an involvement of lobule X in attention and cognitive shifting, in cerebellar disorders (10). Our results of the associations between smaller lobule X volume and non-verbal executive dysfunctions in FEP patients are consistent with the studies mentioned above.

As reviewed in the introduction, and by others (78), inconsistent results have been reported in terms of cerebellar regional alterations in schizophrenia. Nonetheless, when considering only studies that employed cerebellar specific methods, smaller volume in the posterior superior lobe (VI - VIIB) and/or flocculonodular lobe (X) have consistently emerged (19–22). Obviously, clinical heterogeneity could remain a major factor in determining cerebellar structural abnormalities in schizophrenia (25). Nonetheless, we emphasize the importance of avoiding poor alignment of cerebellar structures by employing a cerebellar specific atlas such as SUIT (37), to minimize variance associated with methodology.

Cerebellar structural alterations have been reported mainly from chronic schizophrenia patients (3). However, we suggest that these cerebellar abnormalities are not a result of the adverse effects from a chronic deteriorating course of the illness or antipsychotic medications in patients. Instead, these abnormalities might be present at the early phase of the illness. In the present study, we showed that FEP patients also had cerebellar structural abnormalities, and that these cerebellar changes were not associated with antipsychotic medication effects or illness duration. This was consistent with previous studies that examined FEP and individuals at high risk for psychosis (43, 44). Therefore, we suggest that cerebellar structural alterations are more directly associated with the illness process itself rather than illness chronicity or antipsychotic medication effects in schizophrenia. A longitudinal study should be followed to investigate whether the cerebellar abnormalities are present from very young age in individuals who have a genetic or clinical risk for psychosis.

Our study has some limitations. First, it would be preferable to employ drug-naïve patients to study cerebellar volumes because the cerebellum could be affected by antipsychotic medication (79, 80). Nonetheless, the effect of antipsychotic medication might not be pronounced in our study, because our significant findings remained the same after controlling for medication effects. Second, in each of our participant group, approximately half were males. It is well known that cerebellar structural abnormalities are more pronounced in male than female patients with schizophrenia (26, 60). Hence, it is possible that cerebellar volume deficits in FEP in our study could have been diluted by inclusion of female participants. Further studies with a large sample could investigate cerebellar structural alterations in males and females separately. Finally, we did not strictly control for alcohol consumption in our study. Nonetheless, participants who had a diagnosis of alcohol use disorder were not included in our sample. Given that alcohol abuse is associated with cerebellar atrophy (81), future studies might want to investigate the effect of alcohol on cerebellar volumes even at sub-clinical level.

In conclusion, we provide evidence for smaller volumes in left cerebellar hemispheric lobules VI and X and their association with executive dysfunction in FEP patients. As these regional alterations were also reported in chronic schizophrenia patients using a cerebellar specific template, we conclude that these cerebellar structural abnormalities are related to illness itself, and not to illness progression and/or medication effects in schizophrenia. Whether these cerebellar alterations are associated with a phenotypic marker for emergence of symptoms (e.g., attenuated psychotic symptoms in high-risk individuals) or they are associated with a genetic predisposition to psychosis would need to be determined in future studies.

## Author contributions

TK, K-HL, TL, and JK contributed in drafting and revising the manuscript. JL and JK contributed in collecting the clinical data. TK and KC contributed in acquisition and analysis of MRI data. HO performed neurocognitive functioning tests. All the authors contributed in revising the manuscript.

### Conflict of interest statement

The authors declare that the research was conducted in the absence of any commercial or financial relationships that could be construed as a potential conflict of interest.
