# Supplementary material for: Cerebellar Structural Abnormalities Associated With Cognitive Function in Patients With First-Episode Psychosis
Source: Front Psychiatry. 2018 Jul 3;9:286. doi: 10.3389/fpsyt.2018.00286 (PMC6038730; doi:10.3389/fpsyt.2018.00286)
Supplement: Supplementary file 1 [file Table_1.DOCX]

Table S1. Correlations between volumes of cerebellar lobules and neurocognitive functions in FEP patients

| Lobules | TMT Part A RT | | TMT Part B RT | | RCFT Immediate recall | | RCFT Delayed recall | | COWA Letter | | COWA Category | | WCST Perseverative errors | | WCST Categories completed | |
| --- | --- | --- | --- | --- | --- | --- | --- | --- | --- | --- | --- | --- | --- | --- | --- | --- |
|  | *r* | *p* | *r* | *p* | *r* | *p* | *r* | *p* | *r* | *p* | *r* | *p* | *r* | *p* | *r* | *p* |
| Left hemisphere | | | | | | | | | | | | | | | | |
| I-IV | -0.011 | 0.946 | -0.094 | 0.576 | -0.062 | 0.711 | -0.153 | 0.36 | -0.022 | 0.896 | 0.052 | 0.756 | -0.161 | 0.334 | 0.351 | 0.031 |
| V | 0.117 | 0.484 | 0.055 | 0.745 | -0.375 | 0.02 | -0.329 | 0.044 | -0.235 | 0.156 | -0.094 | 0.575 | -0.226 | 0.172 | 0.231 | 0.164 |
| VI | -0.033 | 0.843 | -0.024 | 0.885 | -0.2 | 0.228 | -0.156 | 0.351 | -0.05 | 0.764 | 0.017 | 0.922 | -0.286 | 0.081 | 0.375 | 0.02 |
| Crus-I | -0.188 | 0.257 | -0.167 | 0.316 | -0.067 | 0.69 | -0.038 | 0.821 | 0.193 | 0.246 | 0.067 | 0.69 | -0.08 | 0.635 | 0.203 | 0.222 |
| Crus-II | -0.286 | 0.082 | -0.387 | 0.016 | 0.304 | 0.063 | 0.337 | 0.039 | 0.318 | 0.052 | 0.332 | 0.042 | -0.15 | 0.367 | 0.317 | 0.053 |
| Vllb | -0.308 | 0.06 | -0.348 | 0.032 | 0.188 | 0.258 | 0.243 | 0.142 | 0.277 | 0.093 | 0.29 | 0.078 | -0.144 | 0.39 | 0.259 | 0.117 |
| Vllla | -0.179 | 0.283 | -0.353 | 0.03 | 0.106 | 0.528 | 0.233 | 0.16 | 0.143 | 0.39 | 0.165 | 0.323 | -0.2 | 0.228 | 0.33 | 0.043 |
| Vlllb | -0.167 | 0.315 | -0.414 | 0.01 | 0.21 | 0.206 | 0.348 | 0.032 | 0.287 | 0.081 | 0.106 | 0.525 | -0.264 | 0.11 | 0.373 | 0.021 |
| IX | -0.358 | 0.027 | -0.362 | 0.025 | 0.189 | 0.257 | 0.252 | 0.127 | 0.307 | 0.061 | 0.216 | 0.192 | -0.17 | 0.307 | 0.355 | 0.029 |
| X | -0.255 | 0.122 | -0.509 | 0.001 | -0.094 | 0.574 | 0.005 | 0.976 | 0.145 | 0.384 | 0.072 | 0.669 | -0.126 | 0.45 | 0.385 | 0.017 |
| Right hemisphere | | | | | | | | | | | | | | | | |
| I-IV | -0.069 | 0.683 | -0.092 | 0.581 | -0.132 | 0.428 | -0.229 | 0.167 | -0.024 | 0.888 | -0.082 | 0.623 | -0.333 | 0.041 | 0.349 | 0.032 |
| V | -0.006 | 0.971 | 0.071 | 0.673 | -0.377 | 0.02 | -0.374 | 0.021 | -0.271 | 0.1 | -0.052 | 0.754 | -0.388 | 0.016 | 0.33 | 0.043 |
| VI | -0.262 | 0.112 | -0.12 | 0.473 | -0.178 | 0.285 | -0.179 | 0.282 | 0.146 | 0.38 | 0.278 | 0.091 | -0.241 | 0.146 | 0.399 | 0.013 |
| Crus-I | -0.327 | 0.045 | -0.3 | 0.067 | 0.064 | 0.703 | 0.124 | 0.46 | 0.223 | 0.178 | 0.095 | 0.571 | -0.164 | 0.324 | 0.368 | 0.023 |
| Crus-II | -0.382 | 0.018 | -0.438 | 0.006 | 0.328 | 0.044 | 0.405 | 0.012 | 0.393 | 0.015 | 0.363 | 0.025 | -0.181 | 0.277 | 0.347 | 0.033 |
| Vllb | -0.26 | 0.116 | -0.401 | 0.013 | 0.257 | 0.12 | 0.353 | 0.03 | 0.41 | 0.011 | 0.303 | 0.064 | -0.106 | 0.525 | 0.263 | 0.11 |
| Vllla | -0.187 | 0.262 | -0.425 | 0.008 | 0.185 | 0.266 | 0.334 | 0.041 | 0.322 | 0.049 | 0.208 | 0.211 | -0.064 | 0.704 | 0.274 | 0.097 |
| Vlllb | -0.174 | 0.295 | -0.53 | 0.001 | 0.19 | 0.254 | 0.291 | 0.076 | 0.341 | 0.036 | 0.151 | 0.367 | -0.089 | 0.596 | 0.36 | 0.027 |
| IX | -0.371 | 0.022 | -0.355 | 0.029 | 0.231 | 0.163 | 0.304 | 0.063 | 0.31 | 0.058 | 0.242 | 0.143 | -0.128 | 0.444 | 0.316 | 0.053 |
| X | -0.134 | 0.423 | -0.305 | 0.062 | -0.012 | 0.945 | 0.092 | 0.584 | 0.25 | 0.129 | 0.008 | 0.963 | -0.145 | 0.386 | 0.396 | 0.014 |
| Vermis | | | | | | | | | | | | | | | | |
| VI | -0.223 | 0.178 | -0.157 | 0.348 | -0.039 | 0.816 | 0.153 | 0.36 | 0.212 | 0.202 | 0.35 | 0.031 | -0.269 | 0.102 | 0.36 | 0.026 |
| Crus-I | -0.012 | 0.943 | -0.234 | 0.158 | 0.168 | 0.313 | 0.27 | 0.101 | 0.292 | 0.075 | 0.053 | 0.75 | -0.076 | 0.648 | -0.069 | 0.679 |
| Crus-II | -0.09 | 0.593 | -0.153 | 0.359 | 0.247 | 0.136 | 0.287 | 0.081 | 0.147 | 0.378 | 0.182 | 0.274 | -0.239 | 0.148 | 0.35 | 0.031 |
| Vllb | -0.384 | 0.017 | -0.073 | 0.665 | 0.064 | 0.702 | 0.055 | 0.741 | 0.076 | 0.652 | 0.105 | 0.532 | -0.032 | 0.848 | 0.177 | 0.288 |
| Vllla | -0.333 | 0.041 | 0.006 | 0.973 | -0.063 | 0.705 | -0.122 | 0.464 | -0.067 | 0.688 | 0.045 | 0.79 | -0.169 | 0.311 | 0.236 | 0.154 |
| Vlllb | -0.254 | 0.125 | -0.123 | 0.463 | -0.053 | 0.752 | -0.129 | 0.44 | -0.037 | 0.828 | 0.083 | 0.62 | -0.22 | 0.184 | 0.228 | 0.168 |
| IX | -0.398 | 0.013 | -0.305 | 0.062 | 0.083 | 0.618 | 0.075 | 0.656 | 0.225 | 0.175 | 0.15 | 0.367 | -0.22 | 0.184 | 0.349 | 0.032 |
| X | -0.125 | 0.454 | -0.148 | 0.376 | 0.028 | 0.867 | 0.02 | 0.906 | 0.058 | 0.731 | 0.063 | 0.706 | -0.016 | 0.926 | 0.077 | 0.648 |

* TMT: Trail Making Test, RCFT: Rey-Osterrieth Complex Figure Test, COWA: Controlled Oral Word Association Test, WCST: Wisconsin Card Sorting Test; RT: reaction time, FEP: first-episode psychosis
