# Supplementary material for: Cerebellar Structural Abnormalities Associated With Cognitive Function in Patients With First-Episode Psychosis
Source: Front Psychiatry. 2018 Jul 3;9:286. doi: 10.3389/fpsyt.2018.00286 (PMC6038730; doi:10.3389/fpsyt.2018.00286)
Supplement: Supplementary file 2 [file Table_2.DOCX]

Table S2. Correlations between volumes of cerebellar lobules and neurocognitive functions in HCs

| Lobules | TMT Part A RT | | TMT Part B RT | | RCFT Immediate recall | | RCFT Delayed recall | | COWA Letter | | COWA Category | | WCST Perseverative errors | | WCST Categories completed | |
| --- | --- | --- | --- | --- | --- | --- | --- | --- | --- | --- | --- | --- | --- | --- | --- | --- |
|  | *r* | *p* | *r* | *p* | *r* | *p* | *r* | *p* | *r* | *p* | *r* | *p* | *r* | *p* | *r* | *p* |
| Left hemisphere | | | | | | | | | | | | | | | | |
| I-IV | -0.312 | 0.064 | 0.032 | 0.854 | 0.036 | 0.839 | 0.145 | 0.413 | 0.16 | 0.365 | 0.127 | 0.475 | -0.064 | 0.7 | 0.09 | 0.584 |
| V | -0.359 | 0.031 | 0.137 | 0.427 | -0.192 | 0.278 | -0.113 | 0.524 | 0.009 | 0.961 | 0.2 | 0.258 | 0.036 | 0.83 | -0.108 | 0.513 |
| VI | -0.397 | 0.016 | -0.036 | 0.834 | -0.012 | 0.946 | 0.066 | 0.71 | 0.108 | 0.543 | -0.009 | 0.961 | -0.059 | 0.722 | -0.093 | 0.573 |
| Crus-I | -0.24 | 0.158 | -0.123 | 0.475 | -0.05 | 0.778 | -0.038 | 0.829 | -0.017 | 0.923 | -0.314 | 0.071 | -0.018 | 0.913 | 0.093 | 0.575 |
| Crus-II | -0.15 | 0.383 | -0.175 | 0.306 | -0.013 | 0.944 | 0.047 | 0.791 | -0.037 | 0.834 | -0.254 | 0.147 | -0.347 | 0.03 | -0.003 | 0.988 |
| Vllb | 0.028 | 0.872 | 0.042 | 0.809 | -0.267 | 0.127 | -0.29 | 0.096 | -0.152 | 0.39 | -0.34 | 0.049 | -0.096 | 0.56 | -0.13 | 0.43 |
| Vllla | 0.063 | 0.716 | 0.006 | 0.974 | -0.322 | 0.063 | -0.34 | 0.049 | 0.03 | 0.865 | -0.294 | 0.091 | -0.129 | 0.435 | 0.003 | 0.986 |
| Vlllb | -0.167 | 0.329 | 0.071 | 0.682 | -0.079 | 0.657 | 0.041 | 0.817 | 0.177 | 0.317 | -0.049 | 0.785 | -0.094 | 0.571 | 0.066 | 0.688 |
| IX | -0.079 | 0.645 | 0.266 | 0.117 | -0.264 | 0.132 | -0.117 | 0.508 | 0.204 | 0.246 | 0.159 | 0.369 | 0.061 | 0.714 | 0.149 | 0.365 |
| X | -0.179 | 0.296 | 0.214 | 0.21 | -0.012 | 0.945 | 0.089 | 0.616 | 0.121 | 0.496 | 0.114 | 0.521 | 0.049 | 0.769 | -0.016 | 0.921 |
| Right hemisphere | | | | | | | | | | | | | | | | |
| I-IV | -0.379 | 0.023 | 0.03 | 0.86 | -0.125 | 0.482 | 0.05 | 0.781 | 0.114 | 0.522 | 0.176 | 0.319 | -0.14 | 0.397 | 0.018 | 0.913 |
| V | -0.424 | 0.01 | 0.127 | 0.459 | -0.244 | 0.163 | -0.12 | 0.5 | 0.038 | 0.833 | 0.169 | 0.34 | -0.021 | 0.899 | -0.003 | 0.985 |
| VI | -0.505 | 0.002 | 0.032 | 0.852 | -0.077 | 0.664 | 0.065 | 0.717 | 0.188 | 0.287 | -0.005 | 0.979 | -0.03 | 0.858 | -0.189 | 0.248 |
| Crus-I | -0.261 | 0.124 | 0.071 | 0.682 | -0.199 | 0.258 | -0.162 | 0.359 | 0.011 | 0.951 | -0.048 | 0.79 | 0.093 | 0.572 | -0.091 | 0.583 |
| Crus-II | -0.155 | 0.366 | -0.231 | 0.174 | -0.04 | 0.821 | 0.052 | 0.771 | -0.022 | 0.901 | -0.101 | 0.57 | -0.149 | 0.367 | -0.038 | 0.819 |
| Vllb | 0.018 | 0.918 | 0.076 | 0.659 | -0.263 | 0.133 | -0.242 | 0.168 | 0.083 | 0.64 | -0.07 | 0.694 | -0.048 | 0.771 | -0.132 | 0.424 |
| Vllla | 0.002 | 0.993 | -0.031 | 0.856 | -0.12 | 0.499 | -0.066 | 0.71 | 0.118 | 0.506 | -0.049 | 0.783 | -0.097 | 0.555 | 0.054 | 0.745 |
| Vlllb | -0.316 | 0.061 | -0.1 | 0.563 | 0.177 | 0.316 | 0.168 | 0.344 | 0.162 | 0.361 | 0.028 | 0.876 | -0.066 | 0.689 | 0.14 | 0.394 |
| IX | -0.051 | 0.768 | 0.299 | 0.077 | -0.185 | 0.296 | -0.115 | 0.519 | 0.167 | 0.344 | 0.024 | 0.894 | 0.03 | 0.854 | 0.15 | 0.362 |
| X | -0.154 | 0.371 | 0.07 | 0.685 | -0.066 | 0.712 | -0.003 | 0.986 | 0.385 | 0.025 | 0.356 | 0.039 | 0.17 | 0.302 | -0.048 | 0.771 |
| Vermis | | | | | | | | | | | | | | | | |
| VI | -0.366 | 0.028 | -0.104 | 0.545 | -0.048 | 0.789 | 0.147 | 0.407 | 0.132 | 0.458 | 0.102 | 0.568 | -0.092 | 0.578 | -0.055 | 0.739 |
| Crus-I | -0.253 | 0.137 | -0.074 | 0.667 | -0.21 | 0.234 | -0.099 | 0.577 | 0.044 | 0.803 | -0.01 | 0.954 | -0.319 | 0.048 | 0.189 | 0.25 |
| Crus-II | 0.088 | 0.609 | 0.317 | 0.059 | -0.204 | 0.248 | -0.23 | 0.19 | -0.048 | 0.787 | 0.086 | 0.628 | 0.039 | 0.815 | -0.083 | 0.615 |
| Vllb | -0.442 | 0.007 | -0.046 | 0.79 | 0.147 | 0.407 | 0.112 | 0.529 | 0.098 | 0.583 | -0.242 | 0.168 | 0.053 | 0.747 | 0.053 | 0.75 |
| Vllla | 0.139 | 0.42 | 0.231 | 0.175 | -0.065 | 0.716 | -0.03 | 0.865 | -0.045 | 0.8 | -0.057 | 0.749 | -0.021 | 0.898 | 0.345 | 0.031 |
| Vlllb | -0.115 | 0.504 | 0.153 | 0.371 | -0.012 | 0.948 | 0.064 | 0.721 | -0.064 | 0.721 | 0.007 | 0.969 | -0.122 | 0.461 | 0.309 | 0.055 |
| IX | -0.262 | 0.123 | 0.149 | 0.385 | 0.022 | 0.902 | 0.128 | 0.472 | 0.111 | 0.531 | 0.058 | 0.744 | -0.022 | 0.893 | 0.193 | 0.24 |
| X | -0.292 | 0.084 | -0.094 | 0.584 | 0.302 | 0.082 | 0.339 | 0.05 | 0.266 | 0.129 | 0.071 | 0.692 | -0.127 | 0.442 | 0.171 | 0.298 |

* TMT: Trail Making Test, RCFT: Rey-Osterrieth Complex Figure Test, COWA: Controlled Oral Word Association Test, WCST: Wisconsin Card Sorting Test; RT: reaction time, FEP: first-episode psychosis
