# Supplementary material for: Cerebellar Structural Abnormalities Associated With Cognitive Function in Patients With First-Episode Psychosis
Source: Front Psychiatry. 2018 Jul 3;9:286. doi: 10.3389/fpsyt.2018.00286 (PMC6038730; doi:10.3389/fpsyt.2018.00286)
Supplement: Supplementary file 3 [file Table_3.DOCX]

Table S3. Group-by-volume interaction effects in the regression model predicting neurocognitive functions

| Lobules | TMT Part A RT | | TMT Part B RT | | RCFT Immediate recall | | RCFT Delayed recall | | COWA Letter | | COWA Category | | WCST Perseverative errors | | WCST Categories completed | |
| --- | --- | --- | --- | --- | --- | --- | --- | --- | --- | --- | --- | --- | --- | --- | --- | --- |
|  | *Beta* | *p* | *Beta* | *p* | *Beta* | *p* | *Beta* | *p* | *Beta* | *p* | *Beta* | *p* | *Beta* | *p* | *Beta* | *p* |
| Left hemisphere | | | | | | | | | | | | | | | | |
| I-IV | 1.71 | 0.271 | -0.57 | 0.723 | -1.25 | 0.504 | -2.47 | 0.176 | -1.32 | 0.44 | -0.20 | 0.898 | -1.08 | 0.522 | 2.63 | 0.11 |
| V | 3.46 | 0.068 | 0.23 | 0.905 | -1.91 | 0.379 | -2.22 | 0.306 | -1.92 | 0.354 | -2.22 | 0.253 | -2.59 | 0.205 | 3.19 | 0.122 |
| VI | 2.42 | 0.288 | -0.02 | 0.994 | -2.41 | 0.392 | -2.63 | 0.342 | -1.67 | 0.517 | 0.30 | 0.9 | -3.94 | 0.1 | 6.02 | 0.012 |
| Crus-I | 0.13 | 0.953 | -1.01 | 0.645 | -0.66 | 0.793 | -0.40 | 0.871 | 1.76 | 0.442 | 4.02 | 0.057 | -0.82 | 0.713 | 1.82 | 0.412 |
| Crus-II | -0.80 | 0.749 | -2.49 | 0.312 | 2.34 | 0.392 | 2.06 | 0.436 | 2.79 | 0.26 | 5.55 | 0.016 | 0.90 | 0.724 | 3.36 | 0.186 |
| Vllb | -2.89 | 0.237 | -3.64 | 0.139 | 4.54 | 0.097 | 5.15 | 0.053 | 3.88 | 0.115 | 6.16 | 0.007 | -0.88 | 0.736 | 3.89 | 0.134 |
| Vllla | -2.31 | 0.345 | -3.94 | 0.102 | 4.58 | 0.084 | 5.77 | 0.025 | 0.87 | 0.718 | 4.44 | 0.047 | -1.58 | 0.527 | 3.67 | 0.138 |
| Vlllb | -0.02 | 0.988 | -3.02 | 0.042 | 1.89 | 0.284 | 1.79 | 0.286 | 0.10 | 0.947 | 0.77 | 0.61 | -1.44 | 0.378 | 2.13 | 0.189 |
| IX | -0.93 | 0.218 | -1.78 | 0.02 | 1.48 | 0.083 | 1.17 | 0.159 | 0.16 | 0.836 | 0.00 | 0.999 | -0.80 | 0.343 | 0.97 | 0.235 |
| X | -0.50 | 0.537 | -2.61 | 0.001 | -0.36 | 0.709 | -0.33 | 0.728 | 0.03 | 0.972 | -0.20 | 0.811 | -0.70 | 0.435 | 1.84 | 0.034 |
| Right hemisphere | | | | | | | | | | | | | | | | |
| I-IV | 1.51 | 0.387 | -0.88 | 0.632 | -0.56 | 0.793 | -2.75 | 0.19 | -1.12 | 0.57 | -1.71 | 0.35 | -3.14 | 0.089 | 3.77 | 0.044 |
| V | 2.78 | 0.185 | 0.63 | 0.772 | -2.23 | 0.354 | -3.37 | 0.157 | -2.97 | 0.194 | -1.76 | 0.415 | -5.07 | 0.021 | 4.41 | 0.051 |
| VI | -0.37 | 0.858 | -1.65 | 0.459 | -1.74 | 0.5 | -2.92 | 0.249 | -0.04 | 0.985 | 2.76 | 0.204 | -3.52 | 0.126 | 6.94 | 0.002 |
| Crus-I | -0.95 | 0.63 | -3.23 | 0.116 | 2.39 | 0.325 | 2.50 | 0.292 | 1.62 | 0.46 | 1.23 | 0.553 | -2.26 | 0.261 | 4.41 | 0.025 |
| Crus-II | -2.00 | 0.345 | -3.04 | 0.147 | 3.47 | 0.147 | 3.13 | 0.172 | 3.05 | 0.155 | 2.97 | 0.143 | -0.98 | 0.67 | 3.49 | 0.121 |
| Vllb | -2.24 | 0.402 | -4.18 | 0.111 | 5.48 | 0.062 | 5.84 | 0.039 | 1.82 | 0.482 | 2.90 | 0.243 | -0.76 | 0.771 | 3.76 | 0.15 |
| Vllla | -1.40 | 0.526 | -3.55 | 0.094 | 2.55 | 0.287 | 3.17 | 0.168 | 0.98 | 0.646 | 1.83 | 0.368 | -0.03 | 0.989 | 2.13 | 0.334 |
| Vlllb | 0.78 | 0.63 | -3.50 | 0.02 | -0.06 | 0.973 | 0.71 | 0.694 | 0.59 | 0.726 | 0.35 | 0.829 | -0.37 | 0.837 | 1.79 | 0.299 |
| IX | -1.44 | 0.108 | -2.32 | 0.012 | 1.68 | 0.097 | 1.71 | 0.081 | 0.52 | 0.567 | 0.71 | 0.411 | -0.73 | 0.457 | 1.20 | 0.214 |
| X | -0.02 | 0.976 | -1.12 | 0.098 | 0.16 | 0.834 | 0.28 | 0.713 | -0.54 | 0.421 | -1.09 | 0.089 | -0.83 | 0.258 | 1.50 | 0.035 |
| Vermis | | | | | | | | | | | | | | | | |
| VI | 0.23 | 0.879 | -0.85 | 0.595 | 0.50 | 0.761 | 1.54 | 0.308 | -2.17 | 0.182 | 3.61 | 0.027 | -0.28 | 0.877 | 0.01 | 0.996 |
| Crus-I | 0.22 | 0.469 | -0.35 | 0.247 | 0.31 | 0.301 | 0.04 | 0.887 | 0.09 | 0.77 | -0.31 | 0.342 | 0.55 | 0.107 | 0.54 | 0.1 |
| Crus-II | -0.62 | 0.599 | -1.46 | 0.222 | 0.89 | 0.438 | 0.70 | 0.515 | -1.65 | 0.171 | 2.68 | 0.026 | 1.85 | 0.142 | 2.23 | 0.069 |
| Vllb | -0.57 | 0.6 | -0.08 | 0.949 | -0.15 | 0.908 | 2.20 | 0.072 | -0.29 | 0.822 | 1.21 | 0.344 | -1.07 | 0.459 | -0.75 | 0.601 |
| Vllla | -2.18 | 0.114 | -0.26 | 0.858 | -0.12 | 0.931 | 1.05 | 0.423 | -1.02 | 0.486 | 0.29 | 0.841 | -0.68 | 0.659 | -1.05 | 0.484 |
| Vlllb | -0.88 | 0.441 | -0.86 | 0.468 | 0.05 | 0.967 | 0.72 | 0.512 | -1.17 | 0.338 | 0.72 | 0.555 | -0.75 | 0.563 | -1.36 | 0.279 |
| IX | -1.29 | 0.245 | -2.27 | 0.055 | 0.62 | 0.593 | 0.57 | 0.603 | -1.48 | 0.242 | 1.75 | 0.159 | 0.16 | 0.905 | -0.28 | 0.824 |
| X | 0.31 | 0.797 | -0.82 | 0.505 | -0.89 | 0.448 | -0.04 | 0.97 | 0.24 | 0.852 | -0.04 | 0.973 | -1.19 | 0.357 | -1.31 | 0.3 |

* TMT: Trail Making Test, RCFT: Rey-Osterrieth Complex Figure Test, COWA: Controlled Oral Word Association Test, WCST: Wisconsin Card Sorting Test; RT: reaction time, FEP: first-episode psychosis

** *Beta*: degree of change in a dependent variable (e.g., neurocognitive function) when a predicting variable (e.g., group-by-volume interaction term) changes one unit.
